# Therapeutic Effects of an Inhibitor of Thioredoxin Reductase on Liver Fibrosis by Inhibiting the Transforming Growth Factor-β1/Smads Pathway

**DOI:** 10.3389/fmolb.2021.690170

**Published:** 2021-09-01

**Authors:** Wenxuan Jiao, Man Bai, Hanwei Yin, Jiayi Liu, Jing Sun, Xiaoxia Su, Huihui Zeng, Jinhua Wen

**Affiliations:** ^1^ State Key Laboratory of Natural and Biomimetic Drugs, School of Pharmaceutical Sciences, Peking University, Beijing, China; ^2^ Department of Cell Biology and Stem Cell Research Center, School of Basic Medical Sciences, Peking University Health Science Center, Beijing, China; ^3^ Shanghai Yuanxi Medicine Corp, Shanghai, China

**Keywords:** liver fibrosis, hepatic stellate cells, thioredoxin reductase, transforming growth factor-β1, collagen, α-SMA

## Abstract

Liver fibrosis is an important stage in the progression of liver injury into cirrhosis or even liver cancer. Hepatic stellate cells (HSCs) are induced by transforming growth factor-β1 (TGF-β1) to produce α-smooth muscle actin (α-SMA) and collagens in liver fibrosis. Butaselen (BS), which was previously synthesized by our group, is an organic selenium compound that exerts antioxidant and tumor cell apoptosis–promoting effects by inhibiting the thioredoxin (Trx)/thioredoxin reductase (TrxR) system. The aim of this study was to investigate the potential effects of BS on liver fibrosis and explore the underlying molecular mechanisms of its action. Liver fibrosis models were established using male BALB/c mice through intraperitoneal injection of CCl_4_. BS was administered orally once daily at a dose of 36, 90, or 180 mg/kg. Silymarin (Si), which is a drug used for patients with nonalcoholic fatty liver disease and nonalcoholic steatohepatitis, was administered at a dose of 30 mg/kg per day as a control. The action mechanisms of BS against liver fibrosis progression were examined in HSCs. The study revealed that the activity and expression levels of TrxR were elevated in the mouse liver and serum after CCl_4_-induced liver fibrosis. Oral administration of BS relieved the pathological state of mice with liver fibrosis, showing significant therapeutic effects against liver fibrosis. Moreover, BS not only induced HSC apoptosis but also inhibited the production of α-SMA and collagens by HSCs by downregulating the TGF-β1 expression and blocking the TGF-β1/Smads pathway. The results of the study indicated that BS inhibited liver fibrosis by regulating the TGF-β1/Smads pathway.

## Introduction

Liver fibrosis is a process of substantial collagen-based deposition of extracellular matrix (ECM) in the liver caused by one or more injury factors, such as alcohol, viral hepatitis, drugs, nonalcoholic steatohepatitis, and autoimmune diseases (Lim and Kim, 2008; Ismail and Pinzani, 2011). In addition, liver fibrosis is a crucial stage in the development of various chronic liver diseases into cirrhosis and even liver cancer. HSCs are the primary fibrogenic cells from the occurrence of liver injury (Puche et al., 2013). Activated by various cytokines, HSCs are continuously transformed into myofibroblasts, in which the TGF-β1/Smads pathway plays an important role. Myofibroblasts increase the expressions of α-SMA, collagen I (Col I), and collagen III (Col III) as well as decrease the activity of the matrix metalloproteinase, which in turn produces a large amount of ECM. Next, fibrous septa and capillaries of the hepatic sinus are formed, eventually leading to liver fibrosis (Brenner, 2009). To date, only few developed medicines have been used in the treatment of liver fibrosis. Although some of them exert antioxidant effects (Zhang and Schuppan, 2014; Latief and Ahmad, 2018), there are no available chemical drugs for the clinical treatment of liver fibrosis.

The thioredoxin system consists of three parts: NADPH, Trx, and TrxR, where TrxR is an NADPH-dependent dimeric selenium enzyme containing a flavin adenine dinucleotide domain (Lu and Holmgren, 2014). There are at least three isoenzymes of TrxR in mammals, namely, TrxR1 in the cytoplasm, TrxR2 in the mitochondria, and TrxR3 in the testis. The TrxR system exerts a direct antioxidant effect and supports the functions of other antioxidant enzymes, protecting cells against oxidative stress caused by exogenous substances or carcinogens. It is a significant system in tumor cells for maintaining homeostasis as well as promoting proliferation and angiogenesis (Bian et al., 2019). The expression and activity levels of the thioredoxin system in the liver may be changed owing to chronic ethanol consumption and other injuries (Shearn et al., 2013). Selenium, which regulates oxidative stress, is an important element in life activities. Selenium compounds (both organic and inorganic selenium) have been found to significantly alleviate liver fibrosis in animal models (He et al., 2004; Ding et al., 2010; Liu et al., 2015). Therefore, our group has independently developed a series of new targeted inhibitors for TrxR containing selenium, such as BS, which have been examined in a prospective study for tumor treatment. The IC_50_ value of TrxR inhibitory activity of BS was 1.03 ± 0.05 μM when DTNB (5,5′-dithiobis-(2-nitrobenzoic acid)) was used as an *in vitro* substrate (He et al., 2012). The study of BS treatment on patients has been registered in the Chinese Clinical Trial Register (Trial Registration Number: CXHL2000685).

In our previous study, we showed that BS, an inhibitor of the Trx/TrxR system, exerts a protective effect against hepatocellular injury, cirrhosis, and hepatocellular carcinoma in mice induced by CCl_4_ (Zheng et al., 2018). BS suppresses the PD-L1 expression through the STAT3 pathway, thus promoting immune responses and inhibiting tumor occurrences (Zou et al., 2018). However, it remains unclear whether the Trx/TrxR system is involved in liver fibrosis progression or whether BS, its inhibitor, attenuates liver fibrosis progression. In the present study, we first found that the expression and activity of TrxR in the mouse liver and serum were significantly increased along with liver fibrosis induced by CCl_4_ injection. Therefore, we aimed to evaluate the effect of BS on liver fibrosis *in vivo* and *in vitro* to elucidate whether BS alleviates liver fibrosis by inhibiting oxidative stress and the TGF-β1/Smads pathway. Silymarin, a herb commonly used for treating liver disease in Europe and Asia (Polyak et al., 2013), was used as a control because its therapeutic effects on liver fibrosis *via* the inhibition of the TGF-β1/Smads pathway have been reported. Our results revealed that the Trx/TrxR system played an important role in the progression of liver fibrosis. As a novel inhibitor of TrxR, BS was proven to have significant therapeutic effects against liver fibrosis in the mouse model, which suggested its potential to be developed into a chemical drug for the treatment of liver fibrosis.

## Materials and Methods

### Chemicals and Reagents

Butaselen (BS) was synthesized in Pharmaron (Beijing, China) and dissolved in DMSO to prepare a 10 mM stock solution. CCl_4_ was obtained from Ouhe (Beijing, China). Olive oil, silymarin, and carboxymethylcellulose sodium (CMC-Na) were purchased from Macklin (Shanghai, China). TGF-β1 was obtained from Novoprotein (Beijing, China). BS was diluted in 0.5% CMC-Na *in vivo* and diluted in dimethyl sulfoxide (DMSO) and phosphate buffer saline (PBS) *in vitro*.

### Cell Culture, Treatments, and Cell Viability Assay

Mouse hepatic stellate cell lines (mHSCs) and normal liver cell line AML12 were provided by Bena cell collection (Henan, China). The mHSCs were cultured in DMEM (Macgene, China), supplemented with 20% fetal bovine serum (Gibco, America), while AML12 was cultured in DMEM/F12 (Hyclone, America) and supplemented with 10% fetal bovine serum, 1% ITS liquid media supplement (Sigma, America), and 40 ng/ml dexamethasone (Solarbio, China). Both cells were maintained at 37°C with 5% CO_2_ and 95% air. 24 h of 5 ng/ml TGF-β1 induction was performed in the mHSCs before BS administration. The sulforhodamine B (SRB) assay was used to measure the cell viability.

### Animals and Treatments

Forty-eight BALB/c mice (six-week-old males) weighing 21–23 g were supplied by the Animal Center, Peking University Health Science Center. The experimental protocols were approved by the Research Ethics Committee of Peking University Health Science Center. Mice were kept in standard environmental conditions and weighed per week. Water and food were freely available.

After 2 days of adaptive feeding, mice were randomized into six groups (8 per group): 1) the control group (NC) (the control group treated with 0.5% CMC-Na), 2) the model group (M) (liver fibrosis induced by CCl_4_), 3) the silymarin group (Si) (the model group treated with silymarin at a dose of 30 mg/kg body weight), 4) the BSL group (BSL) (the model group treated with BS at a dose of 36 mg/kg body weight), 5)the BSM group (BSM) (the model group treated with BS at a dose of 90 mg/kg body weight), and 6) the BSH group (BSH) (the model group treated with BS at a dose of 180 mg/kg body weight). Animals were treated with 25% CCl_4_ in olive oil thrice a week for 8 weeks. After 4 weeks of 25% CCl_4_ intraperitoneal injection, animals were administered BS or silymarin. 2 days after the final CCl_4_ injection, animals were anesthetized using 10% pentobarbital sodium and sacrificed. The liver tissue samples were isolated, and serum was collected for further analysis.

### Serum Biochemistry

Aspartate transaminase (AST), alanine transaminase (ALT), and alkaline phosphatase (ALP) were detected in the Laboratory Animal Department of Peking University Health Science Center. The levels of serum TGF-β1 (Dogesce, DG30107M, China) and TrxR (Dogesce, DG30250M, China) were measured using ELISA according to the manufacturer's instructions.

### Thioredoxin Reductase Activity

The detection of cell TrxR activity was performed using DTNB as per the following description. The working solution was prepared with 2.7720 g of Na_2_HPO_4_·12H_2_O, 0.3526 g of NaH_2_PO_4_·2H_2_O, 0.3722 g of EDTA·2Na, and 20 mg of BSA, dissolved in about 90 ml of deionized water, and adjusted pH to 7.4 and constant volume to 100 ml. The total cell protein was extracted, and its concentration was measured. 30 μg of the protein sample was added (three multiple wells for each concentration) in a 96-well plate, and the volume was made up to 80 μl with the working solution, and incubated at 37°C in an oven for 30 min. 20 μl 5 mM NADPH (the same volume of the working solution was added to the control group) was added, and finally, 100 μl 10 mM DTNB was added. The final determination volume was 200 μl, the final concentration of NADPH was 0.5 mM, and the final concentration of DTNB was 5 mM. The negative control was a mixture of 80 μl working solution, 20 μl NADPH solution, and 100 μl DTNB solution. Immediately, the absorbance value was detected at 405 nm by FlexStation 3 (Molecular Devices). The samples were oscillated for 10 s before the first record, measured once every 15 s, and measured 30 times. The determination time was 450 s. The maximum reaction rate was recorded as the index of enzyme activity. The cell TrxR activity measurements were performed as the means/average of three.

The activity of liver TrxR was measured with ELISA (Crgent Biotech, AE 1964B, China) according to the manufacturer's instructions. Blank wells were set up (the blank control wells did not add samples and enzyme-labeled reagents, and the other steps were the same). Standard product wells and sample wells were set up, and 50 μl of standard products of different concentrations was added to each of the standard product well; 40 μl of the sample diluent was added to the test sample well, and then 10 μl of the test sample was added (the final dilution of the sample was 5-fold). Then, 100 μl of the enzyme-labeled reagent was added to each well, except for blank wells. The plate was covered with the plate cover and incubated at 37°C for 60 min. The solution from the wells was thoroughly aspirated or decanted. The wells were washed 5 times. 100 µl of the working avidin horseradish peroxidase solution was added to each well. The plate was covered with a plate cover and incubated at 37°C for 15 min in darkness. 50 μl stop solution was added to each well to stop the reaction. The optical density of each well was determined within 5 min, using a microplate reader set at 450 nm.

### Apoptosis Assay of HSCs

HSCs were inoculated in 60 mm dishes with a density of 2×10^5^ cells/ml; each dish contained 2.7 ml of cell suspension. Then the dishes were placed in the incubator. After cell adherence overnight, the compound diluent was added according to the specified concentration, and the control group was added with an equal volume of the medium. After a period of time, the cells were recovered and cultured in a 15-ml centrifuge tube, centrifuged at 1,200 rpm for 2 min, discarded the supernatant, and resuspended in 1 ml PBS. Adherent cells in the culture dish were carefully washed twice with PBS precooled at 4°C and digested into single cells by adding 500 ml trypsin. The single-cell suspension was repeatedly blown several times and then transferred into a 1.5-ml EP tube, centrifuged at 1,000 rpm and 4°C for 2 min. The supernatant was discarded and resuspended with PBS. The merged cells were collected and stained with Annexin V/PI (Biosea, China) following the instructions. The samples were examined by flow cytometry.

### Histopathological Examination and Immunohistochemistry

Hematoxylin and eosin (H&E.) staining and Masson's trichrome staining were performed by Servicebio (Wuhan, China). The H&E assessment was performed blindly by an experienced histopathologist as mentioned in Supplementary Table 1. Immunohistochemical analysis with α-SMA (Servicebio, China), Col I (Servicebio, China), and Col III (Servicebio, China) was conducted by the standard procedure. The tissue sections were placed in a repair box filled with EDTA antigen repair buffer (pH 8.0) (Servicebio, G1206) for antigen repair in a microwave oven. Medium and high fire was maintained for 9 min to boil, and the fire was stopped for 7 min for heat preservation, and then transferred to medium and low fire for 7 min. After natural cooling, the slides were placed in PBS (pH 7.4) and shaken on the decolorization shaker 3 times, 5 min each time. The sections were placed in 3% hydrogen peroxide solution and incubated in darkness at room temperature for 25 min. Then, the slides were placed in PBS (pH 7.4) and washed by shaking on the decolorization shaker for 3 times, 5 min each time. The tissue was uniformly covered by 3% BSA (Servicebio, G5001) and sealed at room temperature for 30 min. The sections were dripped with PBS and prepared with the primary antibody in a certain proportion, including α-SMA (Servicebio, GB13044, 1:800), Col I (Servicebio, GB11022-3, 1:800), and Col III (Servicebio, GB111629, 1:500). The sections were laid flat in a wet box and incubated overnight at 4°C. Second antibodies (HRP-labeled) from the corresponding species of the first antibody were added to cover the tissue and incubated at room temperature for 50 min. The color developing time was controlled under the microscope. The positive color was brown-yellow. After retaining the nucleus with hematoxylin, the cells were dehydrated and sealed. The Polaris multispectral tissue imaging quantitative analysis system was used for scanning, and ImageJ software was used for positive area analysis.

### Western Blot Analysis

The protein was extracted and quantified. The samples were subjected to SDS-PAGE and subsequently transferred onto the PVDF membrane (Millipore, America). After being blocked with 5% nonfat-dried milk (Applygen, P1622) for 1.5 h, the membranes were incubated with primary antibodies overnight at 4°C, including TGF-β1 (Novoprotein, CA59), α-SMA (Abcam, ab124964), Col I (Abcam, ab138492), Col III (Proteintech, 22734-1-AP), Smad3 (Abcam, ab40854), p-Smad3 (Abcam, ab52903), Smad2 (Abcam, ab40855), p-Smad2 (Abcam, ab188334), PPAR-γ (Abcam, ab233218), NF-κB p65 (Abcam, ab32536), p-p65 (CST, 3033 T), and TrxR (Proteintech, 11117-1-AP). Appropriate secondary antibodies (Zsbio, ZB2301) were used to incubate with the membranes for 1 h at room temperature. The protein expression, normalized with GAPDH (Proteintech, 10494-1-AP) or β-actin (Bioss, bsm-33139 M) as internal control, was imaged by the ChemiDoc XRS System (BIO-RAD, America). The UniprotkB information of relevant proteins in the manuscript experiment is shown in Supplementary Table 2.

### Statistical Analysis

The one-way ANOVA and two-tailed Student’s t-test were used to analyze the difference between groups using SPSS 20.0 software. *p* < 0.05 was considered statistically significant.

## Results

### Activity and Expression Levels of TrxR Were Elevated in Mice With Hepatic Fibrosis

**FIGURE 1:**
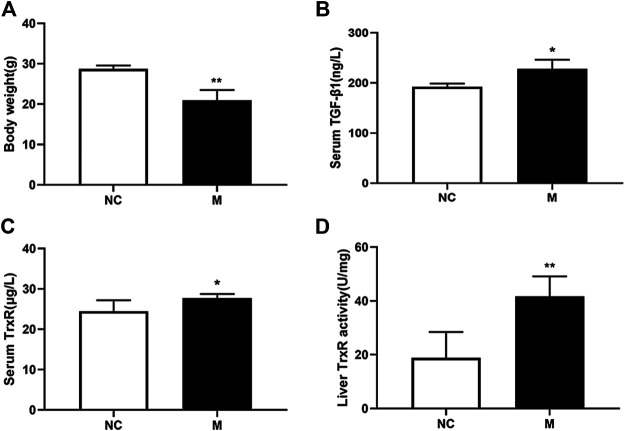
CCl_4_ affected the levels of body weights, TrxR, and TGF-β1 in mouse liver fibrosis models. **(A)** Body weights. **(B, C)** The levels of the liver and serum TrxR were analyzed by ELISA. **(D)** The level of serum TGF-β1 was analyzed by ELISA. Data were shown as mean ± SD, n ≥ 3 and analyzed with two-tailed Student’s t-test. *p < 0.05 and **p < 0.01 in the model group *versus* the control group.

To elucidate whether TrxR is involved in the progression of hepatic fibrosis, mouse models of hepatic fibrosis were established by the intraperitoneal injection of CCl_4_. As shown in Figure 1, in the model group, body weight decreased (Figure 1A), the serum TGF-β1 level slightly increased (Figure 1B), and the serum TrxR expression slightly increased (24.48 ± 2.70 vs. 27.72 ± 1.04 μg/L) (Figure 1C), whereas TrxR activity in liver tissues significantly increased compared to those in the control group (18.80 ± 9.63 vs. 41.69 ± 7.41 U/mg) (Figure 1D). This finding suggested that the activity and expression levels of TrxR were related to the progression of hepatic fibrosis. Therefore, we assessed the efficacy of BS, a newly developed compound, in inhibiting TrxR activity and liver fibrosis.

### The Compound BS Inhibited TrxR Activity

**FIGURE 2:**
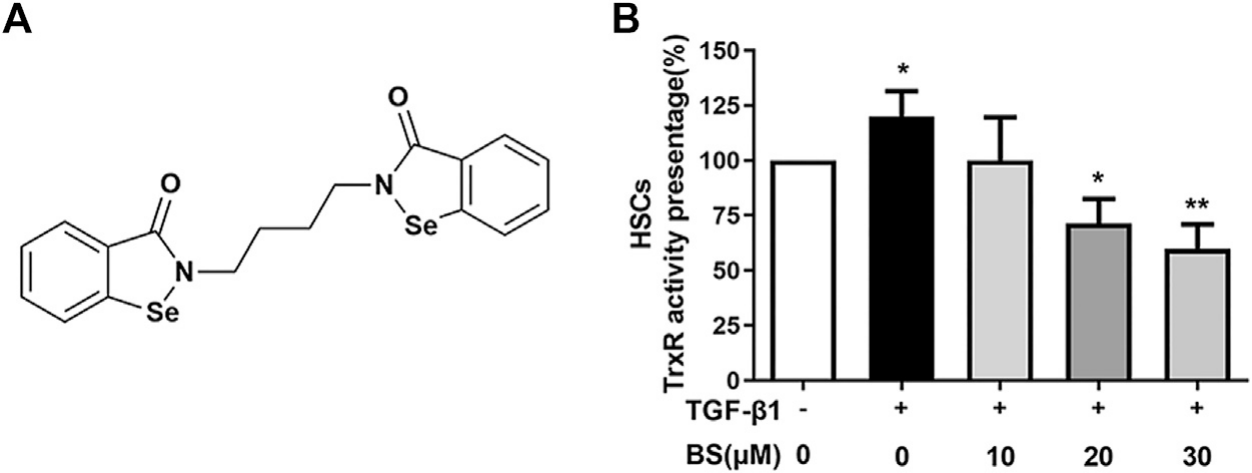
BS decreased TrxR activity in activated HSCs. **(A)** Chemical structure of BS. **(B)** The TrxR activity was assessed in activated HSCs, and the cells were induced by 5 ng/ml TGF-β1. Data were shown as mean ± SD, n ≥ 3 and analyzed with the one-way ANOVA. *p < 0.05, TGF-β1 positive, and 0 μM BS group *versus* TGF-β1 negative, 0 μM BS group. *p < 0.05, **p < 0.01, TGF-β1 positive, 10–30 μM BS groups *versus* TGF-β1 positive, 0 μM BS group.

We detected the TrxR activity–inhibitory effect of BS in liver fibrotic mice. The chemical structure of BS as an organic selenium molecule with symmetrical diselenide rings was shown in Figure 2A. HSCs and the normal liver cell line AML12 were widely used in liver fibrosis studies *in vitro*, and AML12 was used as a control (Li et al., 2019; Yang et al., 2020). The effect of BS was evaluated in an *in vitro* system using HSCs. After HSCs differentiation was induced by TGF-β1, consistent with that in the *in vivo* study (Figure 1D), TrxR activity significantly increased in activated HSCs lysates. However, the TrxR activity was dramatically reduced by BS addition in the culture medium in a dose-dependent manner and recovered to the normal level after 30 µM BS treatment for 48 h (Figure 2B).

### BS Induced Apoptosis of Activated HSCs by Targeting TrxR

**FIGURE 3:**
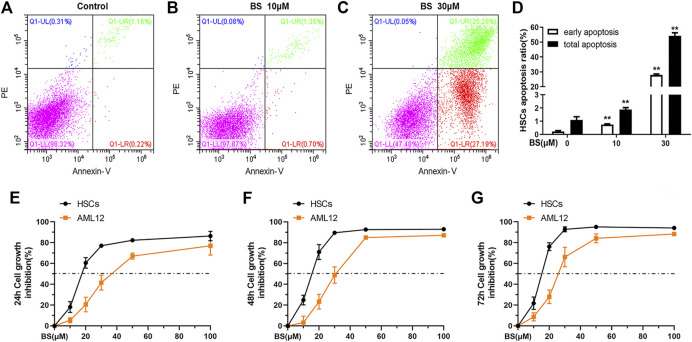
BS induced apoptosis of activated HSCs. **(A–D)** Annexin V-PI analysis and statistical analysis of early apoptosis and total apoptosis rates were presented after BS treatment for 24 h on HSCs. Cell inhibition curves were assessed by the SRB assay under different incubation time of BS on HSCs and AML12 (E: 24 h, F: 48 h, G: 72 h). HSCs were induced by 5 ng/ml TGF-β1. Data were shown as mean ± SD, n ≥ 3 and analyzed with the one-way ANOVA. **p < 0.01, 10–30 μM BS groups *versus* the 0 μM BS group.

BS induces the apoptosis of several lines of TrxR higher expressing hepatocellular carcinoma cells by targeting TrxR (Zheng et al., 2018). Therefore, we investigated whether BS exerts the same effect on TGF-β1–activated HSCs *in vitro*. As shown in Figures 3A–D, both the early and total apoptosis rates of HSCs obviously increased as BS concentration increased. In particular, the early and total apoptosis rates reached 27.91 ± 0.63% and 54.28 ± 2.00%, respectively, after treatment with 30 μM BS.

In addition to inducing apoptosis in activated HSCs, BS inhibited the proliferation of mHSCs and AML12 in a dose- and time-dependent manner. However, the IC_50_ value of BS in activated HSCs was much lower than that in AML12 cells at 24 h (17.95 ± 1.85 vs. 38.38 ± 2.84 μM), 48 h (14.67 ± 0.71 vs. 30.12 ± 1.60 μM), and 72 h (14.40 ± 0.55 vs. 25.83 ± 1.68 μM) (Figures 3E–G). The corresponding HSCs growth inhibition rates were significantly higher in activated HSCs than in AML12 cells after BS treatment for 12 h, 24 h, and 48 h, respectively (Figures 3E–G). These results suggested that BS had a high killing capacity on TGF-β1–activated HSCs by targeting TrxR.

### BS Inhibited the Expressions of Fibrosis-Related Proteins by Regulating the TGF-β1 Pathway

**FIGURE 4:**
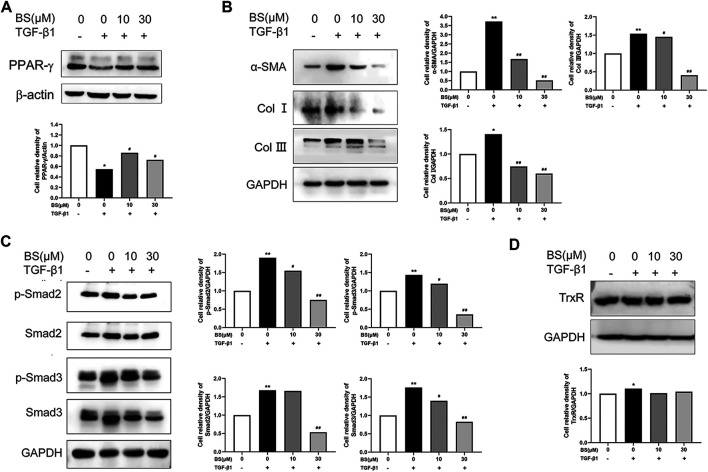
BS inhibited the expressions of fibrosis-related proteins *in vitro*. HSCs were incubated with or without BS and 5 ng/ml TGF-β1. **(A)** The protein levels of PPAR-γ as measured by western blot analysis. **(B)** The protein expressions of α-SMA, Col Ⅰ, and Col III as measured by Western blot analysis. **(C)** The protein expressions of p-Smad2, Smad2, p-Smad3, and Smad3 as measured by Western blot analysis. **(D)** The protein expression of TrxR as measured by Western blot analysis. Data were shown as mean ± SD, n ≥ 3 and analyzed with the one-way ANOVA. *p < 0.05, **p < 0.01, TGF-β1 positive, 0 μM BS groups *versus* TGF-β1 negative, 0 μM BS groups. ^#^ *p* < 0.05, ^##^ *p* < 0.01, TGF-β1 positive, 10–30 μM BS groups *versus* TGF-β1 positive, 0 μM BS groups.

PPAR-γ, a biomarker of quiescent HSCs, is downregulated in activated HSCs (Zardi et al., 2013). Considering that thioredoxin was identified as a target gene of PPAR-α (Liu et al., 2006), we detected the effect of BS on the expression of PPAR-γ in activated HSCs. Our data showed that the PPAR-γ expression was significantly decreased by TGF-β1, but recovered to the normal level in the presence of BS (Figure 4A). This result indicated that BS acted as an inhibitor of HSCs activation by stimulating TGF-β1. Next, we measured the expression of known markers of HSCs activation *in vitro* and hepatic fibrosis *in vivo*, namely, α-SMA, Col I, and Col III, in HSCs after treatment with TGF-β1 in the presence or absence of BS. As shown in Figure 4B, BS significantly downregulated the expressions of α-SMA, Col I, and Col III induced by 5 ng/ml TGF-β1. The phosphorylated and total protein levels of Smad2 and Smad3, two key downstream molecules of the TGF-β1/Smads signaling pathway, decreased in BS in a dose-dependent manner (Figure 4C). Moreover, BS slightly decreased the expression of TrxR in TGF-β1–induced HSCs (Figure 4D). These results indicated that BS prevented HSCs activation or subsequent hepatic fibrosis by inhibiting the TGF-β1/Smads pathway.

### BS Mainly Targeted the Liver and Decreased the Activity and Expression Levels of TrxR in CCl_4_-Induced Hepatic Fibrosis

**FIGURE 5:**
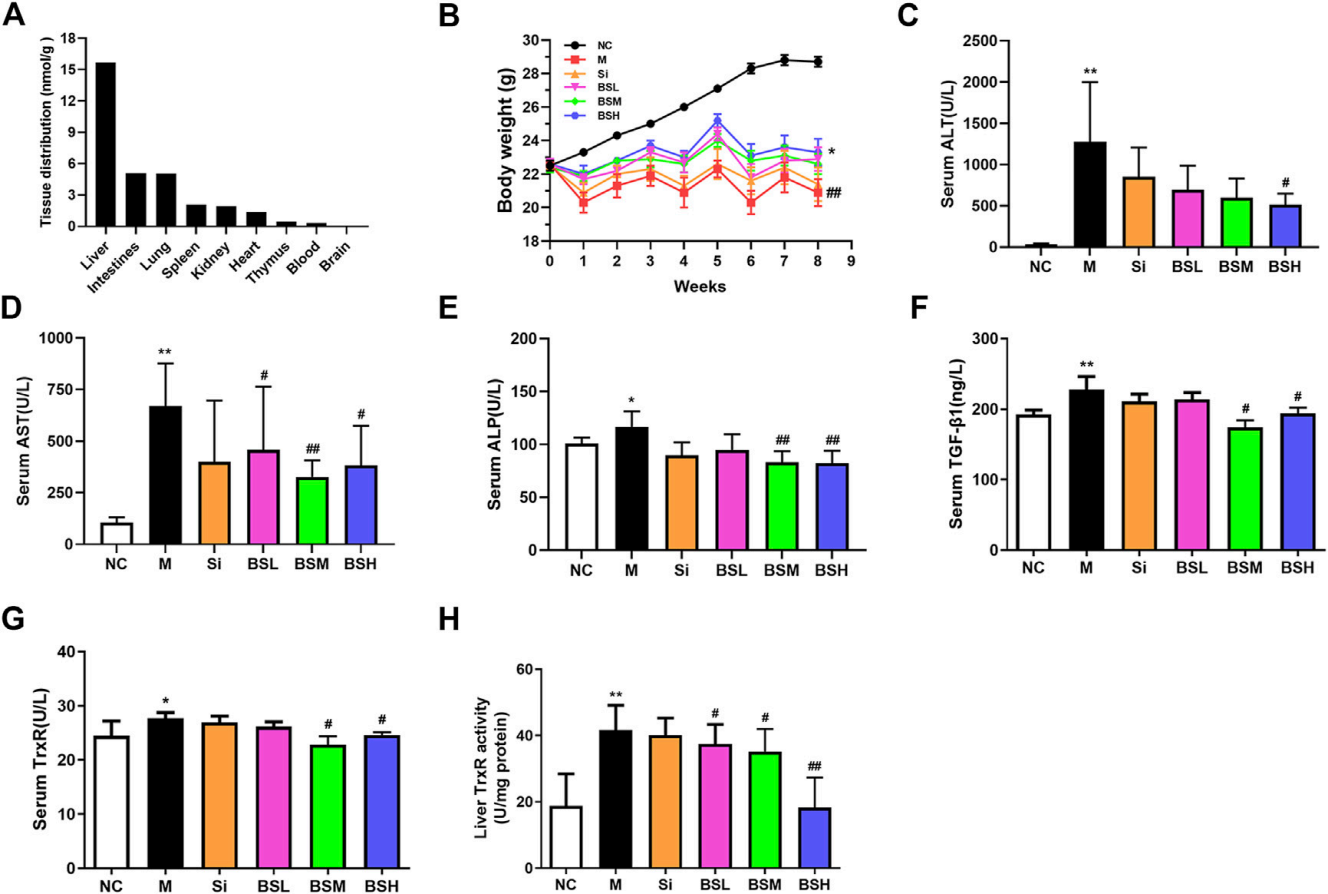
BS ameliorated the biomarkers of CCl_4_-induced liver fibrosis in mice. **(A)** Tissue distributions of BS (180 mg/kg) for 21 days were analyzed. **(B)** Body weight changes. **(C–E)** Liver function was assessed by serum ALT, AST, and ALP. **(F)** Serum TGF-β1 levels. (G–H) The liver and serum TrxR levels were analyzed by ELISA. Data were shown as mean ± SD, n ≥ 3 and analyzed with the one-way ANOVA. *p < 0.05 and **p < 0.01 in the model group *versus* the control group. ^#^ *p* < 0.05, ^##^ *p* < 0.01, Si, BSL, BSM, and BSH groups *versus* the model group.

Before conducting *in vivo* experiments, we investigated the distribution of BS in mouse organs. After administering BS to mice for 21 days, BS was highly accumulated in liver tissues, showing much lower distribution in the intestines, lungs, and other organs (Figure 5A). This finding indicated that BS acted on the liver to inhibit the progression of fibrosis and thus may be beneficial for the treatment of liver fibrosis, cirrhosis, or other chronic liver diseases.

To evaluate the effect of BS treatment on liver fibrosis, an *in vivo* analysis was conducted in CCl_4_-induced liver fibrosis mouse models. Silymarin at the lowest effective dose was selected as a control in this experiment. Considering the toxicity of BS and silymarin, we compared the body weight changes of mice in all treated groups: normal mice and CCl_4_-induced liver fibrosis mice with or without drug treatment. The data showed that BS treatment alleviated the degree of weight loss not only by 9.6, 7.8, and 11.6% in the BSL (36 mg/kg BW), BSM (90 mg/kg BW), and BSH (180 mg/kg BW) groups respectively, compared to that in the model group but also by 2.3% compared to that in the Si group (30 mg/kg BW) (Figure 5B).

Along with the progression of hepatic fibrosis, serum biochemical markers, such as ALT, AST, and ALP, were significantly elevated in the model group mice. The serum levels of these biomarkers were ameliorated in the BS-treated groups: ALT and AST levels were decreased by more than 50%, and the ALP level was decreased by approximately 29% in the BSH group. There was no significant difference between the Si group and the model group (Figures 5C–E). Considering that the TGF-β1/Smads pathway plays an important role in the pathogenesis of liver fibrosis, we detected serum TGF-β1 concentrations in these animal groups, and our results revealed that the serum expression level of TGF-β1 was decreased by more than 23% in the BSM group (Figure 5F). Serum TrxR expression and liver TrxR activity showed similar results to the serum TGF-β1 expression (Figures 5G,H). Moreover, silymarin did not show the same degree of effect as that of BS. This finding suggested that BS prevented hepatic fibrosis development in mice.

### BS Attenuated Liver Fibrosis *In Vivo via* the TGF-β1/Smads Signaling Pathway

**FIGURE 6:**
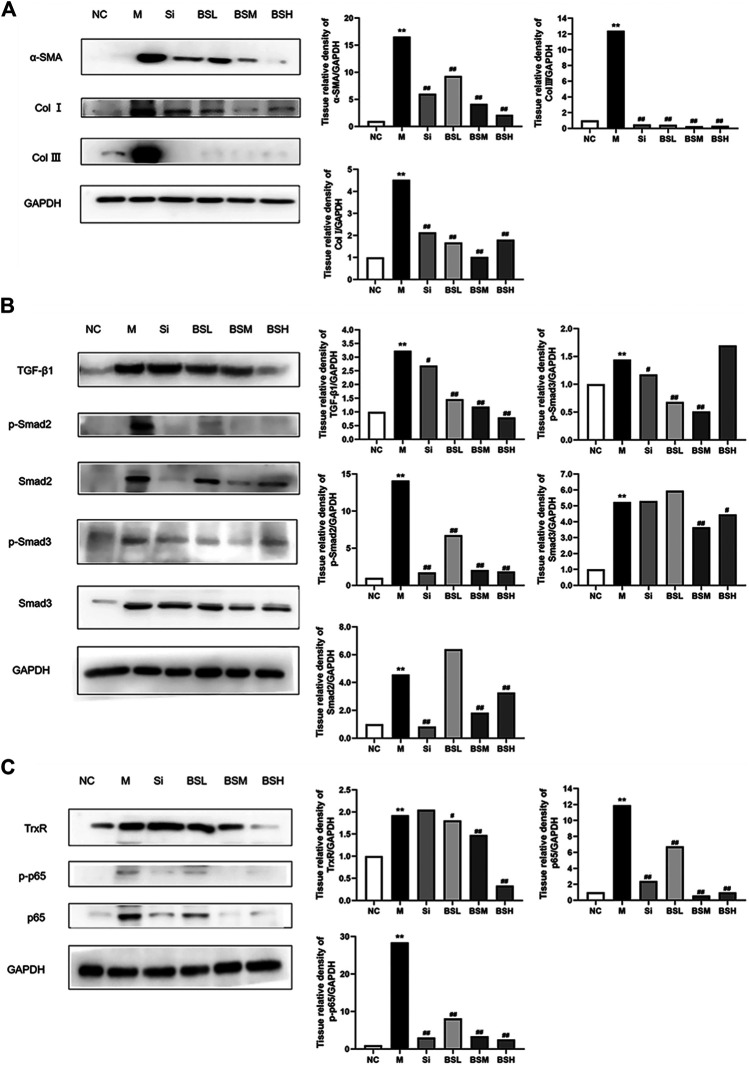
BS inhibited the expressions of fibrosis-related proteins *in vivo*. **(A)** The protein levels of α-SMA, Col I, and Col III as measured by Western blot analysis. **(B)** The protein expressions of TGF-β1, p-Smad2, Smad2, p-Smad3, and Smad3 as measured by Western blot analysis. **(C)** The protein expressions of TrxR, NF-κB p-p65, and p65 as measured by Western blot analysis. Data were shown as mean ± SD, n ≥ 3 and analyzed with the one-way ANOVA. **p < 0.01 in the model group *versus* the control group. ^#^ *p* < 0.05, ^##^ *p* < 0.01, Si, BSL, BSM, and BSH groups *versus* the model group.

To identify the role of BS in liver fibrosis, the effects of silymarin and BS at different dosages on protein expression levels were observed in mouse fibrotic livers by Western blot analysis. The results showed that the expression levels of α-SMA in the liver were attenuated by BS treatment in a dose-dependent manner (Figure 6A). Subsequently, after treatment with BS, a sharp downregulation of Col I and Col III proteins was observed in fibrotic livers compared with that in the model, coinciding with the finding of α-SMA downregulation (Figure 6A). Compared with that in the model group, the TGF-β1 expression was attenuated by BS in a dose-dependent manner, but was reduced to the normal level in the BSH group, showing a greater reduction than that in the Si group (Figure 6B). In addition, the phosphorylated and total protein levels of Smad2 and Smad3 declined significantly in BS-treated livers (Figure 6B). Furthermore, the phosphorylated and total protein levels of Smad2, not Smad3, were decreased in Si-treated livers. The TrxR protein level in mouse fibrotic livers was significantly reduced in the BSH group (Figure 6C). The NF-κB signaling pathways play a central role in oxidative stress, inflammation, and fibrosis. Here, we examined whether the level of phosphorylated/total p-65 was altered by treatment with BS. Western blot analysis revealed that compared with those in the model group, the protein levels of phosphorylated p65 and p65 in mouse fibrotic livers decreased obviously in the BSM and BSH groups, similar to those in the Si group (Figure 6C). Taken together, these results suggested that BS ameliorated the progression of liver fibrosis *via* the TGF-β1/Smads and NF-κB signaling pathways.

### BS Improved the Pathological Characteristics of Fibrosis and Protected the Liver From Fibrosis

**FIGURE 7:**
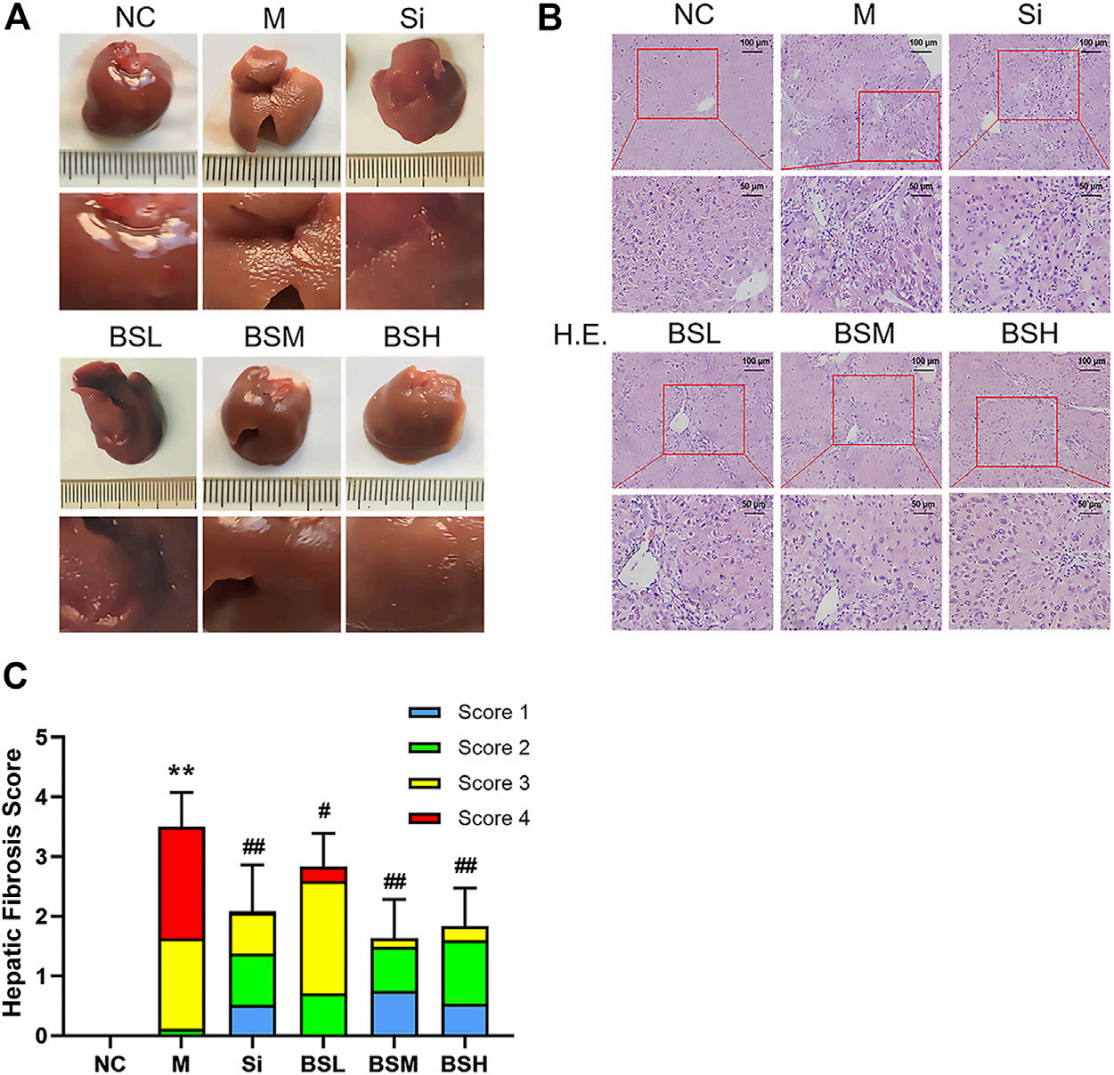
BS improved the pathological characteristics in mouse liver fibrosis models. **(A)** The pathological changes of each group at the end of the experiment. **(B)** Representative liver sections of H&E staining; scale bars = 100 μm, 50 μm as indicated. **(C)** Hepatic fibrosis scores were counted and compared. Data were shown as mean ± SD, n ≥ 3 and analyzed with the one-way ANOVA. **p < 0.01 in the model group *versus* the control group. ^#^ *p* < 0.05, ^##^ *p* < 0.01, Si, BSL, BSM, and BSH groups *versus* the model group.

As shown in Figure 7A, the liver surface of normal mice was smooth, shiny, dark red in color, and soft in texture, showing normal liver appearance. In contrast, the liver surface of the mouse models was rough, frosted, lighter in color, and harder in texture. BS treatment alleviated the above symptoms and greatly restored the normal liver surface appearance. Liver histology was evaluated by H&E staining and histological scoring (see Supplementary Table 3). The results showed the deposition of fibers and severe fibrosis in the H&E–stained liver sections of the CCl_4_-induced model group, with an overall H&E score of approximately 3.5. In contrast, the BS- or silymarin-treated groups showed suppressed fiber deposition and dramatically recovered liver structure (Figure 7B). The scores of the BSM and BSH groups were significantly improved compared with that of the Si group (BSM group: 2.17 ± 0.82, BSH group: 1.71 ± 0.69, vs. Si group: 2.42 ± 0.58) (Figure 7C).

**FIGURE 8:**
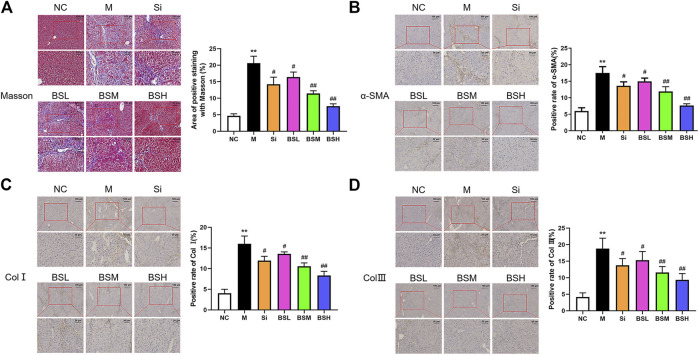
BS ameliorated the fibrosis-related biomarkers *in vivo*. **(A)** Masson's staining was performed on the liver tissues of each group. Representative images were shown. The positive areas of Masson's staining were counted and compared. **(B–D)** Immunohistochemical analyses for α-SMA, Col I, or Col III were conducted on the liver tissues of each group. The positive areas of α-SMA, Col I, and Col III were counted and compared; scale bars = 100 μm, 50 μm as indicated. Data were shown as mean ± SD, n ≥ 3 and analyzed with the one-way ANOVA. **p < 0.01 in the model group *versus* the control group. ^#^ *p* < 0.05, ^##^ *p* < 0.01, Si, BSL, BSM, and BSH groups *versus* the model group.

To complement H&E staining, Masson's staining was conducted to identify collagen (blue) and muscle (red) fibers to determine the fibrosis level (Figure 8A). The findings were similar to those observed with H&E staining. The area positive for Masson's staining in the model group was approximately 4.4-fold higher than that in the normal group, whereas that in the BSH group was much lower than that in the model group (7.58 ± 0.64 vs. 20.62 ± 1.88%).

To further examine the effect of BS on hepatic fibrosis, the expressions and distributions of α-SMA, Col I, and Col III were assessed by immunohistochemical analysis. Compared to that in the model group, the expressions of α-SMA, Col I, and Col III in the BS and Si groups were downregulated, especially in the BSH group. BS decreased the number of α-SMA-, Col I-, and Col III-positive cells around the manifold area in a dose-dependent manner (Figures 8B–D). The area positive for α-SMA, Col I, and Col III was approximately 2.93-, 3.95-, and 4.50-fold larger in the model group than in the normal group. The data showed that BSH (180 mg/kg BW) alleviated the area positive for α-SMA, Col I, and Col III by 56.4, 47.8, and 50.4%, respectively, compared with that in the model group. The decreases in these three parameters in the Si group were lower than those in the BSM group. These results indicated that BS treatment played a therapeutic role against severe hepatic fibrosis by reducing the expressions of fibrosis-related proteins, such as α-SMA, Col I, and Col III.

## Discussion

Liver fibrosis is an integral part of the progression of chronic liver injury to cirrhosis, which can lead to severe liver dysfunction and, ultimately, liver cancer. Currently, no developed medicine has met the clinical challenges in the treatment of liver fibrosis. BS, a compound independently synthesized by our group, is an inhibitor of TrxR activity that exerts antitumor properties through a series of effects, including apoptosis induction (He et al., 2012). The levels of TrxR and Trx are not within the proteins that are most affected in liver cells after the addition of CCl_4_ (Dong et al., 2016), but our previous research indicates that the Trx/TrxR system is likely closely related to the pathology of fibrosis (Zheng et al., 2018). BS inhibits the early occurrence of hepatic carcinoma by inhibiting chronic inflammation and cirrhosis, suggesting its potential efficacy in alleviating fibrosis (Zheng et al., 2018). This study explored the therapeutic effect and molecular mechanism of BS against liver fibrosis.

The TrxR system plays an important role in redox regulation. The relationship between liver fibrosis and the Trx/TrxR system has not been elucidated. This study found, for the first time, that in addition to that of TGF-β1, the expression of TrxR also significantly increased in mice with CCl_4_-induced liver fibrosis. The inhibition of TrxR activity by BS treatment decreased the high level of TGF-β1 and inhibited its signaling pathway. Moreover, BS effectively restored the damaged liver function by suppressing the expressions of fibrosis-related proteins. This result indicated that the Trx/TrxR system was deeply involved in the pathology of liver fibrosis and may be considered as a new target for liver fibrosis treatment.

Located around the hepatic sinusoid, HSCs are key cells in liver fibrosis pathology, accounting for 5–8% of the total hepatocytes (Blaner et al., 2009). After liver injury, resting HSCs are activated and differentiated into myofibrillar-like cells, which are the main cells producing ECM, accompanied by high expression levels of α-SMA, Col I, and Col III (Lee et al., 1995; Hernandez-Gea and Friedman, 2011; Puche et al., 2013; de Oliveira da Silva et al., 2017). An important strategy to treat liver fibrosis is by targeting ECM-producing cells, namely, activated HSCs, in a damaged liver. Promotion of HSC apoptosis and direct inhibition of HSCs activation can alleviate liver fibrosis (Sasaki et al., 2016; Xu et al., 2016; Kikuchi et al., 2017). Our findings showed that BS induced the apoptosis of activated HSCs and blocked their differentiation into ECM-producing cells.

The TGF-β1 signaling pathway plays a key role in cell proliferation and differentiation, and extensive studies have shown that inhibition of this signaling pathway reduces the degree of liver fibrosis (Inagaki and Okazaki, 2007; Verrecchia and Mauviel, 2007; Lichtman et al., 2016). TGF-β1 is a pivotal driver of HSCs activation; it mediates the downstream activation of Smads through TGF-βR. The activated Smads complex is then transported from the cytoplasm to the nucleus, where it acts as a transcription factor and upregulates the expressions of a series of fibrosis-related genes. The role of the Trx/TrxR system on the differentiation of HSCs has long been unknown. In our previous report, BS downregulated the PD-L1 expression of hepatocellular carcinoma cells and inhibited tumorigenesis by blocking the STAT3 pathway in a mouse model (Zou et al., 2018). In this study, we observed that during the differentiation and activation of HSCs *in vitro*, BS not only inhibited TrxR activity but also effectively reduced the expression of TGF-β1 and fibrotic molecules, such as α-SMA, Col I, and Col III. More importantly, in addition to downregulating the expressions of TGF-β1 and TrxR, BS inhibited the activation of the TGF-β1 downstream pathway. It is also important that Trx was reported to regulate Smad3 phosphorylation in ox-LDL–stimulated HUVECs and Trx directly interacted with Smad3/pSmad3 (Chen et al., 2013; Lu Feng, 2018). Several genes related to fibrotic disorders are regulated by reactive oxygen species (ROS) in TGF-β1–induced fibrosis, while ROS can also affect fibrosis through the Smads/non-Smads pathway (Samarakoon et al., 2013). Jiang et al. also reported that Trx and TGF-β1 regulate each other (Jiang et al., 2015). Another study proved that oxidative stress can upregulate TGF-β1 signaling through Smads, while mitochondrial Trx2 can inhibit this phenomenon (Ishikawa et al., 2014). Ethaselen, an analog of BS, could inhibit TGF-β–induced epithelial–mesenchymal transformation in salivary adenoid cystic carcinoma induced *via* snail and slug proteins (Jiang et al., 2015). All of the above studies further supported that the Trx/TrxR system was involved in the TGF-β1/Smads signal pathway to regulate liver fibrosis. It is worth pointing out that the α-SMA expression is related not only to the TGF-β1 signaling pathway (Wang et al., 2012). Since the thioredoxin system may also affect the polymerization state of actin (Lassing et al., 2007), by inhibiting TrxRs, BS must have also depolymerized actin filaments, especially those of α-SMA which belong to the identified interactome of mitochondrial thioredoxin (Chasapis et al., 2019).

In addition, the apoptosis-inducing capacity of BS showed its efficacy in killing activated HSCs. Interestingly, BS exhibited a certain degree of cell-specific killing activity. The degree of BS-induced apoptosis of normal hepatocytes was lower than that of HSCs, which may be caused by the selective targeting of BS on cell types highly expressing TrxR or its differentiating stage. This property of BS may lead to low injury in normal liver tissues during treatment. It is noteworthy that our tissue distribution experiments showed that BS was mainly concentrated in the liver, which indicated that BS targeted abnormal cells in the liver and exerted its physiological effects rapidly after entering the body (Sahi et al., 2010).

Selenium is closely related to the inhibition of liver fibrosis. Selenium supplementation significantly alleviates liver fibrosis in mice and rats by regulating the TGF-β1 pathway or inducing apoptosis (He et al., 2004; Ding et al., 2010; Liu et al., 2015). BS, a TrxR inhibitor, is an organic selenium compound possessing a symmetrical diselenide ring structure with a butyl link arm. Thus, its anti-fibrosis function might be partially attributed to its structure, which contains a selenium molecule.

Silymarin is an extract of milk thistle and is composed of silybin, isosilybin, silydianin, and silychristin. Silymarin has certain anti-fibrotic effects. However, its clinical application is limited owing to poor water solubility, low bioavailability, and side effects such as headache and gastrointestinal effect (Gharbia et al., 2018). Our study found that, unlike silymarin, BS effectively reversed weight loss in mice with CCl_4_-induced hepatic fibrosis. Furthermore, compared with silymarin, BS inhibited the NF-κB, TGF-β1, and other signaling pathways to a greater degree. Further pathological studies showed that BS treatment prevented the liver from entering the stage of cirrhosis, and the therapeutic effects were significantly superior to those of silymarin. Multiple indicators showed that, compared with silymarin, BS can effectively treat liver fibrosis.

In summary, the Trx/TrxR system was revealed, for the first time, to play an important role in the progression of liver fibrosis. Moreover, BS, an inhibitor of this system, exerted significant therapeutic effect on CCl_4_-induced liver fibrosis in mice. BS inhibited the activation of HSCs, thereby attenuating hepatic fibrosis by inhibiting the TGF-β1/Smads signaling pathway. This study suggested that BS may be beneficial in the treatment of liver fibrosis owing to its multiple functions, and that the novel compound is expected to be developed as an effective drug for clinical liver fibrosis treatment.

## Data Availability

The original contributions presented in the study are included in the article/Supplementary Material; further inquiries can be directed to the corresponding authors.

## Abbreviations

BS, butaselen; HSCs, hepatic stellate cells; TrxR, thioredoxin reductase; Trx, thioredoxin; Si, silymarin; TGF-β1, transforming growth factor-β1; ECM, extracellular matrix; α-SMA, α-smooth muscle actin; Col I, collagen I; Col III, collagen III.
