# Supplementary material for: Therapeutic Effects of an Inhibitor of Thioredoxin Reductase on Liver Fibrosis by Inhibiting the Transforming Growth Factor-β1/Smads Pathway
Source: Front Mol Biosci. 2021 Sep 1;8:690170. doi: 10.3389/fmolb.2021.690170 (PMC8440796; doi:10.3389/fmolb.2021.690170)
Supplement: Supplementary file 1 [file Table1.DOCX]

Supplementary Material

**Supplementary Table 1. The Grade Score Criteria of Hepatic Fibrosis.**

| Grade Score | Hepatic fibrosis |
| --- | --- |
| 0 | None |
| 1 | Enlarge in portal area & mild fibrosis |
| 2 | Median fibrosis in portal and periportal area, no bridging fibrosis. |
| 3 | Porto-portal bridging fibrosis, no porto-central bridging fibrosis |
| 4 | Porto-portal and porto-central bridging fibrosis, cirrhosis |
