# Supplementary material for: Therapeutic Effects of an Inhibitor of Thioredoxin Reductase on Liver Fibrosis by Inhibiting the Transforming Growth Factor-β1/Smads Pathway
Source: Front Mol Biosci. 2021 Sep 1;8:690170. doi: 10.3389/fmolb.2021.690170 (PMC8440796; doi:10.3389/fmolb.2021.690170)
Supplement: Supplementary file 2 [file Table2.DOCX]

Supplementary Material

**Supplementary Table 2. The UniprotkB information of relevant proteins in the manuscript experiment is as follows.**

|  | Entry | Entry name | Gene names | Organism | Length |
| --- | --- | --- | --- | --- | --- |
| TrxR1 | Q9JMH6 | TRXR1_MOUSE | Txnrd1 Trxr1 | Mus musculus (Mouse) | 613 |
| PPAR-γ | P37238 | PPARG_MOUSE | Pparg Nr1c3 | Mus musculus (Mouse) | 505 |
| SMAD2 | Q62432 | SMAD2_MOUSE | Smad2 Madh2 Madr2 | Mus musculus (Mouse) | 467 |
| SMAD3 | Q8BUN5 | SMAD3_MOUSE | Smad3 Madh3 | Mus musculus (Mouse) | 425 |
| Collagen 1 | P11087 | CO1A1_MOUSE | Col1a1 Cola1 | Mus musculus (Mouse) | 1453 |
| Collagen 3 | P08121 | CO3A1_MOUSE | Col3a1 | Mus musculus (Mouse) | 1464 |
| α-SMA | P62737 | ACTA_MOUSE | Acta2 Actsa Actvs | Mus musculus (Mouse) | 377 |
| TGF-β1 | P04202 | TGFB1_MOUSE | Tgfb1 | Mus musculus (Mouse) | 390 |
| p65 | Q04207 | TF65_MOUSE | Rela Nfkb3 | Mus musculus (Mouse) | 549 |
