# Supplementary material for: Therapeutic Effects of an Inhibitor of Thioredoxin Reductase on Liver Fibrosis by Inhibiting the Transforming Growth Factor-β1/Smads Pathway
Source: Front Mol Biosci. 2021 Sep 1;8:690170. doi: 10.3389/fmolb.2021.690170 (PMC8440796; doi:10.3389/fmolb.2021.690170)
Supplement: Supplementary file 3 [file Table3.DOCX]

Supplementary Material

**Supplementary Table 3. Distribution of H.E. scores in each group.** Three visual fields of each mouse (n = 8) in each group were selected after H.E. staining, i.e., twenty-four visual fields in each group were scored and the number of score 0-4 in each group was counted.

| Group | Score 0 | Score 1 | Score 2 | Score 3 | Score 4 |
| --- | --- | --- | --- | --- | --- |
| NC | 24/24(100%) | 0 | 0 | 0 | 0 |
| M | 0 | 0 | 1/24(4.1%) | 10/24(41.7%) | 13/24(54.2%) |
| Si | 0 | 6/24(25%) | 10/24(41.7%) | 8/24(33.3%) | 0 |
| BSL | 0 | 0 | 6/24(25%) | 16/24(66.7%) | 2/24(8.3%) |
| BSM | 0 | 11/24(45.8%) | 11/24(45.8%) | 2/24(8.3%) | 0 |
| BSH | 0 | 7/24(29.2%) | 14/24(58.3%) | 3/24(12.5%) | 0 |
